# Supplementary material for: Comparative Transcriptomics Analysis Reveals the Differences in Transcription between Resistant and Susceptible Pepper (Capsicum annuum L.) Varieties in Response to Anthracnose
Source: Plants (Basel). 2024 Feb 15;13(4):527. doi: 10.3390/plants13040527 (PMC10892400; doi:10.3390/plants13040527)
Supplement: Supplementary file 1 [file plants-13-00527-s001.zip › plants-2739106-supplementary-Table.pdf]

# *Supplementary Material*

Article title: **Comparative Transcriptomics Analysis Reveals the Differences in Transcription between Resistant and Susceptible Pepper (*Capsicum annuum* L.) Varieties in Response to Anthracnose**

There are one supplementary table, and the following supplementary information is available for this article:

**Supplementary Table S1 The diameter of the spot after infection with colletotrichum in pepper material**

| Pepper materials | Lesion diameter (cm) | Colletotrichum capsici |
|---|---|---|
| B161 | 1.57 | TJ-3-3 |
| B85 | 1.14 | TJ-3-3 |
| B75 | 1.07 | TJ-3-3 |
| B87 | 0.78 | TJ-3-3 |
| B73 | 1.05 | TJ-3-3 |
| B78 | 1.45 | TJ-3-3 |
| B74 | 0.70 | TJ-3-3 |
| B2 | 0.67 | TJ-3-3 |
| B68 | 0.76 | TJ-3-3 |
| B41 | 0.55 | TJ-3-3 |
| B19 | 0.47 | TJ-3-3 |
| B71 | 0.63 | TJ-3-3 |
| B76 | 0.56 | TJ-3-3 |
| B72 | 0.58 | TJ-3-3 |
| B77 | 0.51 | TJ-3-3 |
| B70 | 0.53 | TJ-3-3 |
| B158 | 0.46 | TJ-3-3 |
