# Supplementary material for: Comparative Transcriptomics Analysis Reveals the Differences in Transcription between Resistant and Susceptible Pepper (Capsicum annuum L.) Varieties in Response to Anthracnose
Source: Plants (Basel). 2024 Feb 15;13(4):527. doi: 10.3390/plants13040527 (PMC10892400; doi:10.3390/plants13040527)

Supplementary Material

Article title: **Comparative Transcriptomics Analysis Reveals the Differences in Transcription between Resistant and Susceptible Pepper (*Capsicum annuum* L.) Varieties in Response to Anthracnose**

There are two supplementary figures, and the following supplementary information is available for this article:

Supplementary Figure S1 All gene expression heat map of phenylpropanoid biosynthesis pathway

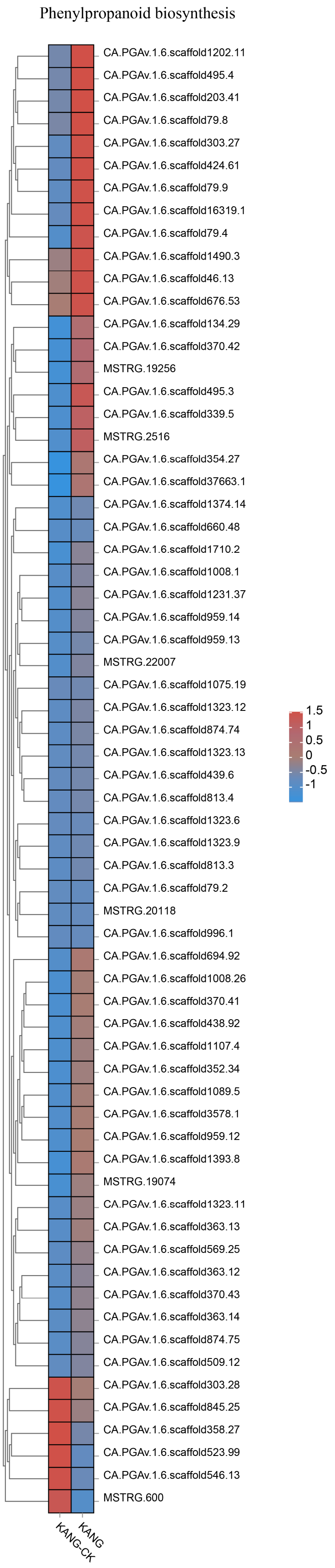

Supplementary Figure S2 All gene expression heat map of plant hormone singal transduction pathway

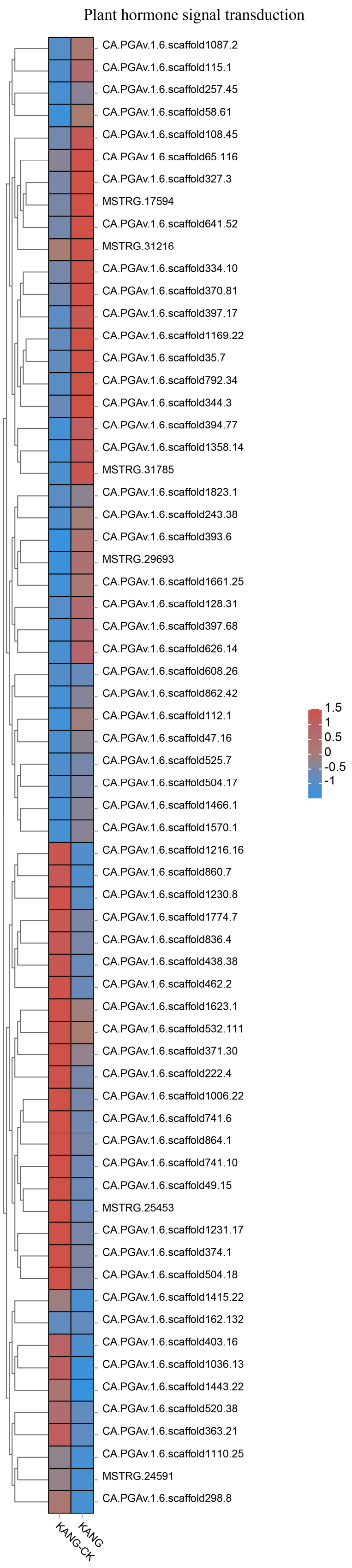

Supplement: Supplementary file 1 [file plants-13-00527-s001.zip › plants-2739106-supplementary.pdf]
